# Simpler Representational Ground

**DOI:** 10.1007/s10670-024-00875-8

**Published:** 2024-10-17

**Authors:** Julio De Rizzo

**Affiliations:** Department of Philosophy, University of Vienna, Vienna, Austria

## Abstract

A common way of clarifying the notion of ground is by way of examples from logic: thus a conjunction is grounded in both of its conjuncts; a disjunction in each of its true disjuncts; a double negation in its negatum; and so on. Developing a semantics that accommodates these logical examples in full generality turned out to be a difficult task. In this paper, I develop a novel approach that substitutes fusion for a more discerning relation of combination between states in the framework of truth-maker semantics.

## Introduction

Recent years have seen a rapidly growing literature on *ground* and *metaphysical explanation* (Raven, 2020). A typical way of introducing the notion goes by way of examples. Though ground is meant to be applicable across the board, more often than not one points to cases from logic: a conjunction obtains, if it does, because both of its conjuncts obtain; a disjunction obtains, if it does, because one of its disjuncts (or maybe both) obtains; a double negation obtains, if it does, because the doubly negated claim obtains; and so on (Correia, 2010; Fine, 2012a, 2012b; Schnieder, 2011).

As once with the notions of necessity and possibility, providing a semantics for ground is a crucial step towards putting the notion on a rigorous footing. In (Fine, 2012a, 2012b) Kit Fine provided a sound and complete semantics for the so-called *pure* logic of ground, that is the logic dealing with principles governing the ground operator leaving aside its interaction with further operators. Fine’s proposal utilizes the framework of truth-maker semantics, which is based on the idea that states, such as that of Socrates’ being wise, *exactly verify* or *make* propositions *true* (Fine, 2017a, 2017b; more on this shortly). Somewhat surprisingly, the proposal can be extended to interpret the *impure* logic of ground, that is the logic dealing with the interaction between ground and truth-functional operators, only at the expense of denying the principles above in their full generality: since the semantics is unable to differentiate between P and P∧P, the former cannot be a ground of the latter, for ground is supposed to be irreflexive; and similarly with P and P∨P; and P and ~  ~ P.

Following Correia (2010, 2020) and Fine (2017b), let us distinguish between *worldly* and *representational* or *conceptual* conceptions of ground. In short, according to the worldly conception, ground tracks *ways reality is*; on the representational conception, ground tracks *how we represent those ways*. The distinction answers to two questions concerning the relata of ground^1^: first, what kind of entities they might be; second, and perhaps more importantly, how fine-grained they are individuated. It is usually assumed that these questions are not independent, that for instance a conception of ground that conceives of P, P∧P, P∨P and ~  ~ P as distinct relata falls on the representational side of the divide (more on this below).

As it turns out, it is not trivial to provide a semantics for representational ground that captures the logical cases we started with. The existing approaches enrich truth-maker semantics in substantial respects: Correia (2017) adds operations on states (verifiers or truth-makers) emulating truth-functional operations at the level of sentences; Krämer (2018) trades truth- and falsity-makers with *modes of verification* and *falsification*, such that ground tracks not only *what* verifies (falsifies), but *how* it does so, and adds an operation of ‘raising’ that takes us to a proposition verified *by way of* verifying the proposition it is applied to; recently deRosset and Fine (2023) provided a semantics that employs two kinds of operation, what they call ‘combination’ and ‘choice’, that operate between sets of states and so-called ‘verification- (and ‘falsification) conditions’, which they distinguish from the latter.

This brief survey is not self-contained, and surely does not pay full justice to the proposals mentioned. But apart from their merits, it should be apparent that they all complicate the picture considerably. It seems a reasonable question whether one could come up with a simpler approach, both with respect to its commitments and the machinery it employs. This question is important for practical purposes, since an approach that allows for a smoother transition from the semantics for worldly ground to the semantics for the representational case would put us in a better position to compare both conceptions. But most importantly, given that the logical cases such a semantics should validate are among the paradigmatic examples of ground, a positive answer would provide a further vindication of the notion in contemporary metaphysics.

In this paper, I provide a simpler truth-maker semantics for representational ground. I start with general considerations on the truth-maker approach and present Fine’s semantics for the logic of worldly ground (§2). I then lay down novel clauses for ground that motivate the present proposal (§3). While there are strong reasons to reject this account with respect to the worldly notion, a closely related proposal turns out satisfactory with respect to the logic of representational ground. In §4, I discuss the latter in detail. I conclude with critical considerations regarding the very distinction between worldly and representational conceptions of ground we started with.

## Worldly Ground and its Semantics

We should first be clear about which cases a logic of ground should capture. A reasonable proposal takes off from what Schnieder calls the ‘core intuition’, namely that sentences governed by truth-functional operators have their truth-values *in virtue of* the truth-values of their component sentences (Schnieder, 2011). With respect to a sentential language equipped with ‘∧’, ‘∨’ and ‘ ~ ’, with the usual truth-functions associated with these, the intuition then motivates the following principles (where ‘ < ’ is the full ground operator, which takes a plurality of sentences on its left-hand side and a single sentence on its right-hand side; cf. Fine, 2012a):

-  <—*Conj.*: P,Q < P∧Q.
-  <—*Disj.* 1: P < P∨Q.
-  <—*Disj.* 2: Q < P∨Q.
-  <—*Double neg.*: P <  ~  ~ P.
-  <—*Neg-disj.*: ~ P, ~ Q <  ~ (P∨Q).
-  <—*Neg-conj.* 1: ~ P <  ~ (P∧Q).
-  <—*Neg-conj.* 2: ~ Q <  ~ (P∧Q).

These are paradigmatic cases employed to introduce the notion of ground: a conjunction holds because its conjuncts do; a disjunction holds because of each of its disjuncts; and so on.

The relevant notion of ground is here *non-factive*: ground might hold between propositions irrespectively of whether they are true. As <—*Conj.* and <—*Neg-disj.* illustrate, the ground operator might take a plurality of sentences on its left side. E.g., a conjunction obtains because both of its conjuncts, *taken together*, obtain. This allows us to express *full* explanations of conjunctions and negations of disjunctions. Following the usual take on this, I assume that ‘ < ’ is asymmetric (hence irreflexive) and transitive.

The first and most discussed semantics of ground was provided by Kit Fine (2012a) in the framework of truth-maker semantics. The core idea of this approach is the relation of *exact verification* between states and propositions (or equivalently for our purposes, sentences expressing these; I will be sloppy about this): the proposition that Socrates is wise, for instance, is verified by the state of Socrates’ being wise; the conjunctive proposition that Socrates is wise ∧ snow is green is verified by the state that is the fusion of Socrates’ being wise and snow’s being green (I will refer to the fusion by ‘Socrates’ being wise ⊔ snow’s being green’). Just as non-factive ground does not require truth of grounds and consequences, verification thus conceived does not require that the verifier obtains. Verification is exact in that every part of the verifier must be relevant for verification. Thus while Socrates’ being wise exactly verifies the proposition that Socrates is wise, Socrates’ being wise ⊔ snow’s being green does not, since it has a part which plays no role in making that proposition true.

Fine also introduces the relation of *exact falsification* between states and propositions. Exact falsification is subject to constraints analogous to the ones just discussed and plays a crucial role in clauses for propositions involving negation. A proposition in this setting is a pair of sets of verifiers and falsifiers.

Relatively to an assignment of verifiers and falsifiers to atomic sentences, the following clauses for truth-functional operators can be stated in this framework (where ‘∣⊢’ and ‘⊣∣’ express exact verification and falsification, respectively; ‘s’, ‘t’ and ‘u’ range over states; universal quantifiers are left implicit):

- s ∣⊢ P∧Q if and only if ∃u∃t (s = t⊔u and t ∣⊢ P and u ∣⊢ Q).
- s ∣⊢ P∨Q iff s ∣⊢ P or s ∣⊢ Q or ∃u∃t (s = t⊔u and t ∣⊢ P and u ∣⊢ Q).
- s ∣⊢ ~ P iff s ⊣∣ P.
- s ⊣∣ P∧Q iff s ⊣∣ P or s ⊣∣ P or ∃u∃t (s = t⊔u and t ⊣∣ P and u ⊣∣ Q).
- s ⊣∣ P∨Q iff ∃u∃t (s = t⊔u and t ⊣∣ P and u ⊣∣ Q).

Notice that the clauses for verification of disjunctions and falsification of conjunctions are *inclusive*, that is, they allow for a fusion of verifiers (falsifiers) to verify (falsify) the disjunction (conjunction). We also suppose that every sentence is verifiable (cf. ‘Verifiability’ in Fine, 2017a). These points will be crucial for the approach presented below.

There are two natural relations of parthood propositions might bear to one another. First, a proposition P might be part of Q whenever every verifier of P *is* a verifier of Q: P, for instance, is a part of P∨Q in this sense. On the other hand, a proposition P might be part of Q whenever every verifier of P is *a mereological proper part of* a verifier of Q: P for instance is a part of P∧Q in this sense (provided Q has a verifier which is not a verifier of P). Taking a clue from the examples, let us call propositional parts in the first sense *disjunctive* and in the second sense *conjunctive* parts.^2^

The semantics for ground developed by Fine (2012a) is based on the relation of disjunctive parthood as defined. Fine employs four distinct notions of ground: *weak full ground* ( ≤), *weak partial ground* (≼), *strict full ground* ( <) and *strict partial ground* (≺). Statements of ground employing these are then spelled out in terms of exact verification as follows (Cf. Fine, 2012a, 72):

- *Weak Full Ground*: P_1_, P_2_, … ≤ Q iff s_1_⊔s_2_⊔… ∣⊢ Q whenever s_1_ ∣⊢ P_1_, s_2_ ∣⊢ P_2_, …;
- *Weak Partial Ground*: P ≼ Q iff P is among some P_1_, P_2_, … such that P_1_, P_2_, … ≤ Q;
- *Strict Full Ground*: P_1_, P_2_, … < Q iff P_1_, P_2_, … ≤ Q and ~ (Q ≼ P), for every P among P_1_, P_2_, …;
- *Strict Partial Ground*: P ≺ Q iff P ≼ Q and *not* Q ≼ P.

That is, a plurality of propositions is a *weak full* ground of a proposition whenever every fusion of verifiers of the former verifies the latter; and a proposition is a *weak partial* ground of another whenever the former takes part in a weak full ground of the latter.^3^ In turn, a plurality of propositions is a *strict full* ground of a proposition whenever they are a weak full ground of the latter; and the latter is not a weak partial ground of any proposition in that plurality. Finally, a proposition is a strict partial ground of another whenever it is a weak partial ground of the latter, while the reverse does not hold.

For illustration of the cases these clauses underwrite, it is helpful to consider cases of ground that do not involve a plurality of propositions as ground. Thus P is a *weak full* ground of Q if and only if every verifier of P is a verifier of Q. (This is disjunctive parthood as defined previously.) P is a *strict full* ground of Q if and only if P is a weak full ground of Q, but Q is not a weak partial ground of P.

As Fine (2012a) points out, this semantics does *not* account for the principles of ground laid down above. For P∧P has the same verifiers (and falsifiers) as P, given that s⊔s = s. Hence P is a weak full ground of P∧P, and P∧P is a weak full ground of P. But then P,P is not a strict full ground of P∧P, against <—*Conj*. For parallel reasons, P is not in general a strict full ground of P∨P, nor are P and ~  ~ P thus related.

These considerations suggest that this semantics captures a notion of ground for which the failures of these distinctions is acceptable: the difference between P and ~  ~ P touching only how they represent the way things are, and not the ways ‘themselves’, one might say that they amount to the same fact ‘out there’.

The idea that a truth-maker semantics along these lines captures the worldly conception of ground is pursued in detail by Correia (2010, 2016). Correia introduces a binary operator of ground-theoretical equivalence ‘≈’—which might be read ‘for it to be the case that … *just is* for it to be the case that…’—to state the admissible ‘identifications’ the worldly ground-theorist might be willing to accept:

*Collapse:*

1. P ≈ P∧P
2. P ≈ P∨P
3. P ≈ ~  ~ P

*Commutativity:*

1. P∧Q ≈ Q∧P
2. P∨Q ≈ Q∨P

*Associativity:*

1. P∧(Q∧R) ≈ (P∧Q)∧R
2. P∨(Q∨R) ≈ (P∨Q)∨R

*Distributivity:*

1. P∧(Q∨R) ≈ (P∧Q)∨(P∧R)
2. P∨(Q∧R) ≈ (P∨Q)∧(P∨R)

*De Morgan:*

1.  ~ (P∧Q) ≈ (~ P∨ ~ Q)
2.  ~ (P∨Q) ≈ (~ P ∧ ~ Q)

Now while *Collapse* (that is, the principles therein) and *Commutativity* are arguably necessary for a conception of ground to earn the ‘worldly’ title, the other principles are open for debate. As a matter of fact, there are compelling reasons to give up *Distributivity* 2. (Krämer & Roski 2015, Correia, 2016). For the following scheme has only true instances: P,Q < (P∨Q)∧(P∨R). By *Distributivity 2*, it follows that P,Q < P∨(Q∧R), given that the groundees in both cases are ground-theoretically equivalent. But consider the case where P is ‘Someone other than Fine is an American philosopher’; Q is ‘Fine is a philosopher’; and R is ‘Fine is American’. Then the result of instantiating these claims in the scheme results in the claim that someone other than Fine is an American Philosopher ∨ Fine is an American philosopher *because*, in part, Fine is a philosopher. But this is implausible: Fine’s being a philosopher does not help bring about the former truth in any way (Krämer & Roski 2015: 63).

## An Alternative Proposal

Weak full ground is the core notion in Fine’s clauses for a semantics for ground. As laid down above, it amounts to a form of disjunctive parthood at the level of verifiers and falsifiers: if propositions are sets of verifiers, it amounts to set-theoretical inclusion of propositions. To recall, a proposition is a conjunctive part of another if and only if every verifier of the former is a proper part of a verifier of the latter. While disjunctive parthood rests on *set-theoretical inclusion* between propositions, conjunctive parthood rests on *mereological proper inclusion* between their members, that is their verifiers and falsifiers.

Fine starts with disjunctive parthood to define the strict notions. The following proposal begins with conjunctive parthood instead. We use ‘⊑’ and ‘⊏’ for improper and proper parthood among states, respectively, defined as usual.^4^ For convenience, let us have a symbol for a relation of conjunctive parthood between propositions^5^:

- *Conjunctive Parthood*. P ⊂ _c_ Q ↔ _df_ ∀s∃t (s ∣⊢ P → (t ∣⊢ Q ∧ s⊏t)).

The clauses underlying the present proposal then read:

- Strict Partial Ground*: P_1_, P_2_, …≺ Q iff for every P among P_1_, P_2_, …: P ⊂ _c_ Q and ~ (Q ⊂ _c_ P).
- Strict Full Ground*: P_1_, P_2_, … < Q iff P_1_, P_2_, …≺ Q and s_1_⊔s_2_⊔… ∣⊢ Q whenever s_1_ ∣⊢ P_1_, s_2_ ∣⊢P_2_, …

(In the spirit of generality, we allow for pluralities to be partial grounds: under the present approach, the operator ‘≺’ is also many-one. Fine’s approach can easily be modified to accommodate this.) In English: a plurality of propositions is a strict partial ground* of another whenever every proposition among the former is a conjunctive part of the latter, and the reverse does not hold; and a plurality of propositions is a strict full ground* of another whenever they are a strict partial ground* of the latter; and every fusion of verifiers of the propositions in the plurality verifies the latter.

As apparent from the principles concerning ‘ < ’ and truth-functional operators above, ground is supposed to track logical complexity: in general, consequences are not logically simpler than their grounds (Schnieder, 2011).^6^ Both approaches follow this thread: while Fine allows for widening of the set of verifiers *and* ‘widening’—that is, mereological extensions—of the verifiers themselves, the present approach allows only the latter.

How does this proposal compare to Fine’s? Without the resort to proper mereological parthood, the current proposal would simply be a notational variant of Fine’s. Crucially, it is more restrictive: every ground-theoretical claim underwritten by the present approach is a ground-theoretical claim underwritten by Fine’s, but not vice-versa.^7^ Thus, for instance, P∧Q < P∨(P∧Q) holds (provided P and Q are not ground-theoretically equivalent) in Fine’s semantics; but it does *not* in the present approach. For, if s the sole verifier of P and t the sole verifier of Q, then s⊔t is *not* properly contained in s or in itself. Therefore, not even the principle that disjunctions are grounded in their disjuncts comes out valid in this semantics.^8^ Moreover, this semantics also validates claims of the form. P,Q ≺ P∨(Q∧R), which caused trouble for *Distributivity* 2.

These consequences show that the present approach is *not* tenable for worldly ground. However, not all is lost: this approach might be developed into an interestingly simpler semantics for the representational case, as we shall now see.

## Simpler Representational Ground

A semantics should specify what features of content ground tracks, i.e. is sensitive to; and what connection between those contents must obtain for grounding to hold between them (deRosset & Fine, 2023). In the worldly case, Fine’s approach gives informative answers to both questions: the set of verifiers or truth-makers of a proposition is tracked by ground; and the relation is formally specified for each case of ground as above.

In this section, I develop a novel semantics for the representational case based on the alternative approach of the previous section. Part of its interest comes from the fact that the modifications from which it results are ‘minimally invasive’ with respect to Fine’s original semantics: indeed, to such an extent that the very same answers to the questions posed to the semantics in the more coarse-grained case might be carried over to the present context. Importantly, I do not claim that these modifications are *better* philosophically motivated than those underlying the alternative approaches mentioned earlier. To present the proposal as a simpler alternative, it suffices that these modifications are *at least equally* philosophically motivated, which I hope is clear from a comparison of the approaches.^9^

As in the worldly case, a logic of representational ground might be characterized by which principles of ground-theoretical equivalence it underwrites. Every logic of representational ground should not only deny the principles in *Collapse* but validate their negations. (Only then might the schemes for ‘ < ’, in a language with ‘∧’, ‘∨’ and ‘ ~ ’, hold unrestrictedly.) But the other families of principles are up for discussion.

In usual truth-maker semantics, the operation of mereological fusion is used to generate states. Fusion is idempotent:

⊔—*Idempotency*: s⊑t → s⊔t = t.

That is, a state fused with one of its parts is simply the state itself.^10^

Ours proposal starts out with a simple move with far-reaching consequences: we substitute fusion by a distinctive operation on states called *combination*, which we symbolize by ‘ + ’ (the terminology follows deRosset & Fine, 2023). Crucially, combination is not only *non*-idempotent, but satisfies instead:

 + *Alterpotency:* s⊑t → (s + t) ≠ t.

(We define parthood in terms of ‘ + ’ below; for a first grip on + *Alterpotency*, the usual notion will do.) Accordingly, applications of combination must result in a different state, even when a state is combined with one of its parts. I will suppose that ‘ + ’ operates on *finite sequences* of states and obeys the following principles (Fine, 2010: 573)^11^:

*Leveling*: + (…, + (s_1_, …, s_n_),…, + (t_1_,…,t_m_),…) =  + ( s_1_, …, s_n_, …, t_1_,…,t_m_).

*Permutation*: + ( s_1_, s_2,_…, s_n_) =  + (s_n_, s_1_, s_2_, …, s_n-1_) [and so on, for every permutation].

(*A note on notation*: To avoid overload, I keep with the notation in terms of lists; I ask the reader to bear in mind that, in the context of combination, the list stands for the corresponding finite sequence, not the plurality^12^; for simplicity, I will assume sequences of states are themselves states, a state in the previous sense being identified with its single-term sequence.)

According to *Leveling*, combination is not sensitive to the combination of ‘inner’ states. According to *Permutation*, combination is not sensitive to differences in the ordering of states in the sequences to which it applies.

Since the clauses of ground from the previous section employ the notion of conjunctive parthood, we should define a notion of part between states by means of the notion of combination. Let x be a *component* of y whenever y is the result of applying ‘ + ’ to x or x together in a sequence with other states (we allow for 1-term sequences). That is: x is a *component* of y iff ∃z (x is a term in the sequence of states z and + (z) = y) (cf. Fine, 2010: 567). Next we define the notion of *being a mereological part* as the ancestral of *being a component*: x is an (improper) part of y (x⊑_+_y) iff x = y or there is a sequence of states 〈x_1,…,_ x_n_〉 such that for every i (1 ≤ i ≤ n), x_i_ is a component of x_i+1_ and x = x_1_ and y = x_n_. In turn, a state x is a proper part of y (‘x⊏_+_y’) if and only if x ≠ y; and there is a sequence of states 〈x_1,…,_ x_n_〉 such that for every i (1 < i ≤ n), x_i_ is a component of x_i+1_ and x = x_1_ and y = x_n_. We note that the relations of mereological parthood are partial orders (weak and strict, respectively).^13^

The clauses for verification and falsification of sentences governed by truth-functional connectives should then be modified with combination in place of fusion. Importantly, since + (s) and + (s,s) are distinct, we include combinations of single states in the clauses. The clauses for conjunction and disjunction become (negation will be treated separately shortly):

1. **Conjunction Verification:**

s ∣⊢ P∧Q if and only if i) ∃t (s=+(t) and t ∣⊢ P and t ∣⊢ Q); *or* ii) ∃u∃t (s=+(t,u) and t ∣⊢ P and u ∣⊢ Q);

That is, a state verifies a conjunction whenever it is the combination of a state which verifies both conjuncts; or of states each of which verifies a conjunct.

2. **Conjunction Falsification:**

s ⊣∣ P∧Q iff i) s ⊣∣ P; *or* ii) s ⊣∣ Q; *or* iii) ∃t (s=+(t) and t ⊣∣ P or t ⊣∣ Q); or iv) ∃u∃t (s=+(t,u) and t ⊣∣ P and u ⊣∣ Q);

That is, a state falsifies a conjunction whenever it falsifies either conjunct; or is a combination of a single state that does; or is a combination of states each of which falsifies a conjunct.

3. **Disjunction Verification**

s ∣⊢ P∨Q iff i) s ∣⊢ P; *or* ii) s ∣⊢ Q; *or* iii) ∃t (s=+(t) and t ∣⊢ P or t ∣⊢ Q); or iv) ∃u∃t (s=+(t,u) and t ∣⊢ P and u ∣⊢ Q);

That is, a state verifies a disjunction whenever it verifies either disjunct; or is a combination of a single state that does; or is a combination of states each of which verifies a disjunct.

4. **Disjunction Falsification**

s ⊣∣ P∨Q iff i) ∃t (s=+(t) and t ⊣∣ P and t ⊣∣ Q); or ii) ∃u∃t (s=+(t,u) and t ⊣∣ P and u ⊣∣ Q);

That is, a state falsifies a disjunction whenever it is a combination of a single state which falsifies both disjuncts; or is a combination of states each of which falsifies a disjunct.

Henceforth, ‘I⊢’ and ‘⊣I’ express these novel notions of verification and falsification. We understand conjunctive parthood with ‘⊏_+_’ in place of ‘⊏’ (for space, I leave this formulation aside). The clauses for ground now are:

- Strict Partial Ground* P_1_, P_2_, …≺ Q iff for every P among P_1_, P_2_, …: P ⊂ _c_ Q and *not* Q ⊂ _c_ P.
- Strict Full Ground*: P_1_, P_2_, … < Q iff P_1_, P_2_, …≺ Q and + (s_1_, s_2_,…) ∣⊢ Q whenever s_1_ ∣⊢ P_1_, s_2_ ∣⊢ P_2_, …

(The English rendering would have ‘combination’ instead of ‘fusion’ only.)

Finally, to read these clauses, we should be clearer on the adicity of combination (or better: the length of the sequences to which combination might apply). Are there boundaries to how many (occurrences of) states are allowed in sequences ‘ + ’ is applicable to? For simplicity and since it has no bearing on most applications in the context of ground, we set the case of no states (0) aside.^14^ But in the spirit of generality, we allow for combination to apply to any arbitrary non-empty *finite* sequence of states (see note 11). This is not only convenient as it touches the formalism, but is also a reasonable choice given that fixing on any specific length for the sequences would face the charge of arbitrariness. Notice that s⊏_+_ + (s) and + (s) ⊏_+_ + (s,s), since by *Leveling* + (s,s) =  + (+ (s), + (s)).^15^

A first noteworthy feature of this approach is that these clauses make properties of combination correspond directly to the first three families of principles of ground-theoretical equivalence above. Thus *Collapse* and its denial (that is, the denial of each principle therein) correspond to + *Idempotency* and + *Alterpotency*, respectively; *Commutativity* and *Associativity* stand or fall with the homonymous properties of combination.^16^ In the simplest version I shall consider, commutativity and associativity of combination *hold*—these follow from *Permutation* and *Leveling*, respectively –, so that *Commutativity* and *Associativity* also hold. But more fine-grained conceptions might be obtained under the present proposal by dropping these requirements.

Under a plausible assumption on the space of propositions, we may now distinguish between P∧P, P∨P and P in terms of their verifiers. Call a set of states S *unbounded* iff for every state s ∈ S, there is a t ∈ S such that s⊏_+_t. Say that a proposition is unbounded whenever its set of verifiers or its set of falsifiers is unbounded in the previous sense. The assumption is, then, that no proposition is unbounded. That is, every proposition is such that in its set of verifiers, some state is not properly contained in any further verifier (the same for falsifiers).^17^ Using this, we now have:

1. P < P∨P.
2. P, P < P∨P.
3. P, P < P∧P,

as required from the examples we started the paper with.^18^

The following amendment addresses the behavior of negated statements. In the worldly setting, P and ~ P ‘swap’ their verifiers and falsifiers: s is a verifier of P if and only if it is a falsifier of ~ P, and vice-versa. We adopt the clause for falsification without modification, but now require that unary and binary combinations of falsifiers of P be included as verifiers of ~ P:

5. Negation Verification

s ∣⊢ ~P iff s ⊣∣ P; or ∃t such that t ⊣∣ P and s=+(t); or ∃t∃u such that t ⊣∣ P and u ⊣∣ P and +(t, u)= s;

That is, a state verifies a negation whenever it either falsifies the negatum; or is the combination of a state that does; or is a combination of states that do.

6. Negation Falsification

s ⊣∣ ~ P iff s ∣⊢ P.

That is, a state falsifies a negation whenever it verifies its negatum.

From which it follows, under the same assumption above^19^:

4) P <  ~  ~ P.
5) P, P <  ~  ~ P.

(Interestingly, Krämer (2018) and deRosset and Fine (2023) also propose an asymmetric treatment of verifiers and falsifiers of negated statements. Barred further differences in detail, their accounts adopt the opposite view to the one just outlined: while I propose to distinguish between verifiers of a negation and falsifiers of the negatum, and identify falsifiers of a negation with verifiers of the negatum, they propose to distinguish between falsifiers of a negation and verifiers of the negatum; and to identify verifiers of a negation and falsifiers of the negatum.)

How could one motivate this somewhat idiosyncratic treatment of negation? A first motivation is that it captures the grounds of double-negated statements in full generality, as just mentioned. Since we let repetition bear on the individuation of for instance P∧P and P∨P, a natural suggestion by way of motivation would be to find a connective that somehow ‘corresponds’ to the operation we use at the level of states. *Peirce’s arrow* (‘↓’) is just such a connective. For ‘P↓Q’ is true if and only if neither ‘P’ nor ‘Q’ are true. P↓P is then truth-functionally equivalent to ~ P. But these claims might differ in terms of their verifiers: in analogy to the negation of disjunctions, P↓P is plausibly verified by either a falsifier of P taken alone, or combinations of falsifiers of P. This condition is close enough to the operation at the level of states we are assigning to ‘ ~ ’ on the representational conception. A language equipped with Peirce’s arrow, thus, would be in a sense more congenial to the present approach, as it would make the ‘binary’ treatment of negation reasonably parallel to those of conjunction and disjunction.^20^

Aside from the failure of *Collapse*, the present proposal validates *Associativity* and *Commutativity*. In contrast, *Distributivity* and *De Morgan* fail. For the first, notice that P∧(Q∨R) has as a verifier + (s,t,u), for s,t and u verifiers of P,Q and R, respectively. But this combination does *not* verify (P∧Q)∨(P∧R). Instead of the ground-theoretical equivalences in *Distributivity* 1*.*, we have the following full ground claim:

- P∧(Q∨R) < (P∧Q)∨(P∧R).

(Given that every verifier of the first is properly included in a verifier of the second, and the converse does not hold.) An analogous line of reasoning shows that *Distributivity* 2. fails.

For *De Morgan*, consider ~ P∨ ~ Q. For simplicity, say that s and t are the sole verifiers of P and Q, respectively; and -s and -t the sole falsifiers of P and Q, respectively (‘-’ is not a sign for any operation on states, but just a device for exposition). Then the set of verifiers of ~ P is {-s, + (-s), s + (-s,-s)}; and the set of verifiers of ~ Q is {-t, + (-t), + (-t, -t)}. By the clause for verification of disjunctions, the set of verifiers of ~ P∨ ~ Q is {-s, -t, + (-s), + (-t), + (-s,-t), + (-s,-s), + (-t,-t)}, that is the union of verifiers of ~ P and ~ Q together with unary combinations of falsifiers of either and the combination of the falsifier of P and the falsifier of Q. On the other hand, the set of verifiers of ~ (P∧Q) under the same assumptions is {-s, -t, + (-s), + (-t), + (-s,-t), + (-s,-s), + (-t,-t), + (-s,-t,-s,-t)}, that is the set having as members falsifiers of P, falsifiers of Q, unary combinations thereof, the combination of the falsifier of P and the falsifier of Q—which falsify P∧Q –, *plus* the binary combination + (-s + −t, -s + -t), which by *Leveling* is identical to + (-s,-t,-s,-t). Hence the sets of verifiers for each of these claims are different. Since every verifier of ~ P∨ ~ Q is a proper part of the last state mentioned, we also have it that:

-  ~ P∨ ~ Q <  ~ (P∧Q).
-  ~ P∧ ~ Q <  ~ (P∨Q).

The resulting notion of ground is thus sensitive to the scope of negation, where complex propositions governing negated simpler sentences ground negated complex propositions ‘containing’ these sentences.

On this approach, truth-maker frames are pairs 〈S, + 〉, where S is a non-empty set and + is a combination operator on S. Frames in the representational and in the worldly case are thus structurally alike (cf. Fine, 2012b), which makes it easier to compare both approaches from a model-theoretical perspective (for reasons of space, I do not undertake this task here).^21^

Finally, notice that the implementation of combination is largely independent of the semantic clauses for ground in terms of conjunctive parthood. That is, one could in principle stick to the original Finean clauses for ground with combination in place of the usual fusion with analogous results. However, I take it that the clauses provide an intuitive underpinning for the role combination is meant to play in ground-theoretical statements. Without the availability of those alternative clauses, the mere substitution of combination for fusion would seem but an ad hoc, purely formal exercise.

### More on Combination

As stated, combination is a variably polyadic operation on states taken to conform to some principles, such as *Alterpotency*, *Leveling* and *Permutation*. Could one provide a more detailed, principled story in favor of combination thus characterized?

Bennett (2013) outlines a mereology that allows for things to be parts of objects ‘twice over’ (see Cotnoir, 2015 for a formal development of a mereology along related lines). According to Bennett, parthood is analogous to other relations in this respect:

The same relation can hold multiple times between the very same entities at the very same time. The cousin of relation, for example, can hold twice between the same two individuals. Two people are cousins twice over, or ‘double cousins’, as they are called, just in case they are the children of pairs of siblings. (83)

The core idea in Bennett’s account is to distinguish between *occupants* and *slots* they occupy. A mereological whole might be conceived of as a set of pairs of slots and occupants. While it is allowed that one occupant occupies more than one slot, each slot has only one occupant.

The contrast with views that accept idempotency for fusion is best illustrated by an example. Consider simple distinct objects a, b, and c. The fusion of a and b overlaps with the fusion of b and c. Take now the fusion of these two fusions. How many simple proper parts does it have? Standard extensional mereology answers: three. An account without idempotency might answer: four. That is, we might count b as a part *twice over*. According to this alternative picture, fusion adds to mereological structure, whereas on the standard view, mereological structure in this sense might be lost by fusing states. Let *a*, *b* and *c* be pictures of Bob, Mick and Peter. In both mereologies, fusing the first two yields a picture of Bob and Mick, and fusing the latter two yields a picture of Mick and Peter. But fusing these two latter pictures again in the standard sense yields a picture of Bob, Mick and Peter, each represented once; while in the non-idempotent sense we end up with a picture including Mick’s image twice.

One must be more precise about the idea of slots and occupants for it to work for our purposes. Conceiving of matters in these terms naturally invites one to resort to considerations from debates on *relational complexes*. In short, a relational complex is an entity that results from the application of a relation (or property) to some relata (Fine, 2000). If combination encodes information about which occupants occupy which slots, one might end up having to distinguish between s + t and t + s: in the first complex, s occupies the slot that t occupies in the second one, whereas t occupies in the first complex the slot that s occupies in the second one. Hence the resulting complexes are distinct. However, this line of reasoning relies on an individuation of *slots* we need not buy into. Granted, if slots are thought of in analogy with spatial locations, reversing occupants that way plausibly results in a change of complexes (even in strictly symmetrical spaces, if one relies on a prior differentiation between spatial locations). But resorting to this conception to allow for one and the same object occupying two distinct slots is at the very least unattractive to someone willing to drop idempotency.^22^

Slots might also be conceived of as *roles*. Say that *a* is mother of *b* and mother of *c*. Then *a occupies* or *plays* the role of *mother-of-b* and of *mother-of-c*.^23^ In the present context, this view might indeed identify s + t and t + s: if s is distinct from t, s occupies the role of being combined with t in both cases. On the other hand, s + s might be distinguished from s by the fact that in the first complex, s plays the role of being combined with itself, whereas in the latter it does not. These desirable features aside, I doubt that talk of roles in this context is illuminating. For first, no reason is given for why roles are not more discerning than we’ve been supposing: what favours *being-combined-with-t* in comparison with, for instance, *being-combined-with-t-on-its-left* as the role played by s in s + t? Besides, while complexes with relations such as *being a mother of* might be cashed out in these terms, transposing role-talk to parts in general seems doubtful as a way of motivation of the features we are trying to capture.

If idempotency should fail and commutativity and associativity should hold, resort to slots is thus of little help. Borrowing once again from the debate on relational complexes, a more promising route is the following. Consider the relations of *being parallel to* and of *being between*. There is a clear sense in which we would *not* want to distinguish between the complexes in the following pairs: *a*’s being parallel to *b* and *b*’s being parallel to *a*; and *b*’s being between *a* and *c* and *b*’s being between* c* and *a*. Being strictly symmetric, *a*’s being parallel to *b just is b*’s being parallel to *a*, and similarly with the other case. The same, one might suggest, with combination: combining states s and t delivers one and only one entity, namely the fusion of them, which might be *referred to* either by ‘s + t’ or by ‘t + s’. Thus the analogy with strictly symmetric relational complexes provides initial support for *Permutation*.

With respect to relational complexes, the application of a reflexive relation to an entity should be different from the entity itself. That is, *a* is distinct from *a*’*s being self-identical* (that is, the complex of *a* instantiating self-identity), and distinct from *a*’s *being the same height as a*. Furthermore, *a*’s *being self-identical* being self-identical—that is, the state being identical with itself—is also distinct from *a*’s self-identity. Keeping with the analogy, the result of combining *a* with itself is distinct from *a*, just as the application of a reflexive relation to an object results in a distinct complex. This analogy thus supports + *Alterpotency* above.

Associativity of combination, or *Leveling* more generally, is less obviously motivated along these lines. Is *a*’s being identical to (*b’s being identical to c*) the same as the relational complex of *c*’s being identical to (*a*’s *being identical to b*)? The following simple argument shows that it is not. Say that *b* and *c* are identical and *a* is the complex of *b*’s being identical to *c*. Then the relational complex of *a*’s being identical to (*b*’s being identical to *c*) obtains, whereas *c*’s being identical to (*a*’s being identical to *b*) does not. Therefore, associativity must fail with respect to relational complexes in general.

To restore the analogy, one might respond to this difficulty in either of two ways. First, one might drop + *Associativity* (cf. Krämer (2018, 2019); Sect. 4.2 below; this would make combination structurally like multi-set formation operation, if taken as variably polyadic).

Alternatively, one might interpret the variable (finite) polyadicity of combination in a distinctive way. Consider, for instance, the properties expressed by the predicates ‘form a set’, or ‘form a galaxy’, which are arguably variably polyadic. Successive applications of these are distinctive in the sense that ‘larger’ complexes thus generated do not fully incorporate ‘embedded’ ones: {a,b} is the result of applying the property of *forming a set* to a,b; and {c,d} is thus related to c,d; but the fact that a,b,c,d form a set is *not* the same as the fact that {a,b},{c,d} form a set. The same with galaxies: galaxies *within a galaxy* are not just the galaxy formed by all the stars involved (cf. the Hoag’s object). The situation is different with e.g. ‘play music together’: if George and John, who play music together, also play music together with Paul and Ringo, who also play music together, the resulting complex is the same as George, John, Paul and Ringo playing music together; or set union, if variably polyadic: the union of (a,b,c,d) is simply the union of the union of (a,b) and the union of (b,c). In such cases, the larger application incorporates the smaller ones. Combination might then be interpreted along the lines of the latter examples, which makes it conform to *Leveling*.

Wrapping up these considerations: the distinction between slots and occupants brings more difficulties than it solves when it comes to motivating the acceptance of *alterpotency*, *commutativity* and *associativity* of combination. A more promising view is to conceive of combined states as formed by parts and a *strictly symmetric* operation of combination which is variably polyadic (thus symmetric in every of its ‘argument-places’, meaning that permutations preserve identity of the fused state). The strict symmetry accounts for *Permutation*, hence commutativity. Finally, the variable polyadicity of combination, interpreted in a distinctive way which makes larger combinations ‘outweigh’ embedded ones, accounts for *Leveling*, hence associativity. Though no doubt inconclusive, these analogies give a better grip on what is at stake and provides for a prima facie case for combination as a legitimate operation to be incorporated in the framework.^24^

### Comparison with Extant Accounts

In the following, I will briefly outline three approaches to the semantics of representational ground, namely that of Correia (2017), Krämer (2018) and deRosset and Fine (2023). The aim of this brief survey is to enable a comparison with the present proposal, not to argue in favor of or against any of them. A table showing how each of the families of principles for ground-theoretical equivalence fares in each of the accounts is displayed at the end of this subsection.

#### Correia (2017)

Correia (2017) adopts Fine’s clauses for ground with slight adaptations and develops a provably sound and complete semantics for a representational logic of ground. He introduces operations on states mirroring operations expressed by the connectives in the object language. Besides a ‘conjunctive’ operation (fusion), there is thus a disjunctive operation and a negation operation on states. He requires that sentential letters have atomic propositions as their semantic values, that is only atomic states as verifiers and falsifiers.

While of undeniable interest, especially in view of the soundness and completeness results, no illuminating answer is forthcoming to the question of what grounding is sensitive to on this approach: the exact same hierarchical structure we see in formulas is reflected at the level of propositional content. Correia is aware of this, and characterizes propositions functionally as ‘the items that play the role of the relata of the relation of strict grounding’ (Correia, 2017: 515). In addition, the commitment to the atomicity of propositions expressed by sentential letters is a limitation to be stressed.

Nonetheless, Correia’s approach agrees with ours with respect to every of the principles of ground-theoretical equivalence above: it invalidates not only *Collapse*, but also *Distributivity* and *De Morgan*; while it validates *Commutativity* and *Associativity*. His approach, however, does *not* underwrite the grounding claims corresponding to these principles, such as (~ P∨ ~ Q) <  ~ (P∧Q); and (~ P∧ ~ Q) <  ~ (P∨Q). Hence even though we individuate propositions alike, ground is treated importantly differently in both approaches.^25^

#### Krämer (2018, 2019)

Krämer’s (2018) proposal starts off with the idea that ground is sensitive not only to *which* states verify which statements, but to *how* statements are thus verified, that is their *mode of verification* (similarly with falsification). For instance, a disjunction P∨Q might be verified *by means of* or via verifying P, or *by means of* verifying Q or *by means of* verifying both (2018: 799). The semantic content of a statement is given by its *modes of verification* and *falsification*. Hence P∨P will be distinguished from P itself, since one might verify the former *by means of* verifying the latter, while this is not the case with P. With respect to conjunction, the reading of the ‘by’ locution is non-distributive, that is, in effect a fusion of states s⊔t verifies P∧Q by means of having a part that verifies P and having a part that verifies Q (and not by verifying P and verifying Q, as it would be on a distributive reading). P and P∧P are then distinguished accordingly. Lastly, for negation, one must consider both modes of verification and modes of falsification. Krämer favors a view according to which ~ P is verified the way P is falsified, that is the modes of verification of ~ P and of falsification of P are the same; whereas ~ P is falsified *by means of* verifying P (Krämer, 2018: section 4). Krämer mode-ifies Fine’s clauses for ground above and defines full and partial strict ground directly in terms of modes of verification (ibid. section 5).

To construe the hierarchy of modes Krämer’s account relies on, he then introduces an operation of *raising* on propositions. For a proposition P, ↑P—the result of ‘raising’ P—is then the proposition that is verified via P and any modes of verification of P itself.

Krämer’s approach provides an interesting account of what features of content ground is sensitive to. Interestingly, different conditions on the assignments of semantic values are shown to correspond to different granularity conditions on ground-theoretical content (Krämer, 2018: section 5.5; Krämer, 2019).

Even under the simpler account, Krämer’s approach invalidates *Associativity* principles. It also validates *De Morgan* equivalences, even under more stringent requirements on the assignments. These two features set Krämer’s approach aside from the present proposal with respect to ground-theoretical equivalence.

#### DeRosset and Fine (2023)

As a starting point, deRosset & Fine distinguish between *content* and *truth* and *falsity-conditions*. As I understand it, this distinction in effect allows them to construe a fine-grained account of ground in the semantics without the need to change the picture of what truth- and falsity-makers *are*. That is, the complexity needed to account for a more fine-grained individuation of propositions is not located at the level of the sets of verifiers and falsifiers of a statement, which make up its content. In addition to *what is* required by a statement for it to obtain, the truth condition encodes the additional information as to *how* the verifiers must be given for the statement to obtain (similarly for falsity conditions and falsifiers). Though distinct, there is a two-way route from contents to conditions: operations on contents yield truth and falsity conditions, and the pairing of truth and falsity conditions yield contents.

DeRosset & Fine introduce an operation on contents they call ‘combination’, which plays a role parallel to fusion in the worldly semantics. Remarkably, combination is neither idempotent, nor associative nor commutative in their account. As a result, commutativity and associativity for conjunction are given up. Say that S is the set of verifiers of P and T the set of verifiers of Q. Then the set of verifiers of P∧Q will be the combination of S and T, while the verifier of Q∧P will be the combination of T with S, which are distinct truth-conditions.

Commutativity and associativity of conjunction should stand or fall with the corresponding equivalences for disjunction. To distinguish between P∨Q and Q∨P, deRosset & Fine introduce a further operation on contents they call ‘choice’. As with combination, this operation is sensitive to order: the choice between contents S and T need not be the same as the choice between T and S.

As in Krämer’s account, the negation ~ P of a proposition P has as verifiers the falsifiers of P; while the set of falsifiers of ~ P is given by the ‘raised’ set of verifiers of P, that is, ~ P is falsified by means of P being verified. *Raising* is identified with combination applied to a single item, which is in turn the same as the result of the application of *choice* to that item.

DeRosset & Fine present a detailed picture of what is required of propositions for them to stand in a ground-theoretical connection. To state the corresponding clauses for ground, they finally make use of a relation at the level of semantic content they call ‘selection’. They prove soundness and completeness with respect to a suitable representational logic of ground.

The account is informative with respect to the content ground is sensitive to and surely interesting in its own right. The individuation of content it offers is notoriously *very* fine-grained: *none* of the principles of ground-theoretical equivalence above holds on their account. Moreover, as in the present account, the account preserves the core idea that grounding tracks mainly verifiers, or truth-makers, of propositions. However, it trades this off against a comparatively high degree of complexity resulting from the introduction of different operations on truth- and falsity-conditions, which again though different from states, have a direct bearing on how propositions are made up from them; plus the commitment to not only combination, but selection as a primitive semantic notion. The present proposal is simpler than theirs also in this regard.

The following table provides an overview of the comparison of the accounts discussed with respect to the principles concerning ground-theoretical equivalence.

**Table:** 

Families of principles	Correia (2017)	Krämer (2018)	deRosset and Fine (2023)	Present proposal
*Collapse*: P ≈ P∧P P ≈ P∨P P ≈ ~ ~ P	✕	✕	✕	✕
*Commutativity:* P∧Q ≈ Q∧P P∨Q ≈ Q∨P	✓	✓ (under the simple account)	✕	✓
*Associativity*: P∧(Q∧R) ≈ (P∧Q)∧R P∨(Q∨R) ≈ (P∨Q)∨R	✓	✕	✕	✓
*Distributivity*: P∧(Q∨R) ≈ (P∧Q)∨(P∧R) P∨(Q∧R) ≈ (P∨Q)∧(P∨R)	✕	✕	✕	✕
*De Morgan:* ~ (P∧Q) ≈ ~ (P∨ ~ Q) ~ (P∨Q) ≈ ~ (P∧ ~ Q)	✕	✓	✕	✕

## Conclusion

Contemporary analytic metaphysics is marked by a growing interest in hyperintensional notions, such as grounding, essence, fundamentality, laws, and others. Spelling these out formally requires more discerning tools for individuating propositions than allowed, for instance, by possible worlds semantics.

In a series of recent papers, Kit Fine developed a semantic framework that allows one to differentiate between necessarily equivalent statements in terms of their *verifiers* and *falsifiers*, that is states-of-affairs that exactly verify and states that exactly falsify them, respectively.

If one conceives of propositions as sets of verifiers and falsifiers, there are at least two natural notions of parthood that might hold between propositions: the verifiers of P might be verifiers of Q; or the verifiers of P might be *proper mereological parts* of verifiers of Q. Fine (2012a) starts off with the former notion when providing a semantics for ground. In this paper, I outlined a view that takes the latter instead as a starting point. The account is more restrictive than Fine’s with respect to the logic of worldly ground, that is the logic that calls for a more coarse-grained individuation of grounds and groundees. But it points to a version of the semantics for the representational logic of ground which is significantly simpler than existing accounts: in particular, it only calls for different constraints imposed on the notion of mereological combination between states, which plays a role analogous to fusion in the semantics for worldly ground.

As it often happens, simplicity comes with a price: the account is in a further sense restricted since it is committed, for instance, to the inclusive treatment of the verifiers of disjunctions (and to the inclusive treatment of the falsification of conjunctions). That is, it must allow for fusions of verifiers of both disjuncts to verify their disjunction. Otherwise, it cannot account for the claim that a disjunction is grounded in each of its disjuncts.^26^ More generally, the account requires that sentences governed by a connective are verified by at least one combined state larger than the verifiers of the embedded sentences. Although flexibility is surely a virtue for semantics in general, it is debatable whether a semantics for ground *should* allow for non-inclusive disjunctions and similar cases. Given the naturalness of the current account and its simpler approach to handling representational ground, I believe that this shortcoming does not disqualify it as a viable contender.

Let me conclude by posing a natural question not addressed thus far: what should we take states *to be* on the present proposal? Recall that the distinction between worldly and representational conceptions of ground is supposed to be sensitive to both the individuation—how fine-grained ground-theoretical equivalence is—*and* the nature of the relata—that is, whether they are *worldly* or *representational* entities of some sort. Although in the proposal presented here the resulting individuation of the relata of ground falls on the representational side of the divide, I do not see any compelling reason not to conceive of states as worldly items, even if formed by combination as an operation of a more discerning sort. Put in other terms, it does not seem unreasonable to hold *both* that states are parts of the world ‘out there’ *and* that combination generates mereological structure in every combined state, where combination is conceived along the lines just discussed.

Correia (2016, 2020) makes use of the notion of *descriptive equivalence* between sentences to characterize ‘wordliness’ of entities designated by nominalization of sentences, for instance ‘the state that p’, ‘the fact that p’, and the like. Following Correia, let us use square brackets applied to a sentence for the former designator. Then the category of *states* is worldly if and only if for all sentences ‘p’ and ‘q’ (of any language) that correspond to states: if ‘p’ and ‘q’ are descriptively equivalent, then [p] and [q] are identical (2020: 232). Descriptive equivalence is in turn defined as follows: ‘p’ and ‘q’ ‘are descriptively equivalent if and only if they describe the world as being the same way’ (230). Setting aside concerns about quantifying over ‘all languages,’ even if these characterizations offer some added clarity, I’m not sure they help in resolving specific cases (and I don’t believe Correia would argue otherwise). For someone holding that [~ ~ p] is different from [p] would presumably also hold that ‘ ~  ~ p’ and ‘p’ are *not* descriptively equivalent in the sense defined. The dispute would now turn on the individuation of ‘ways’ the world might be.

In answer to a possible objection, Dorr (2016: 62–3; cf. Correia 2020: note 17) sketches an argument that might be interpreted as an argument for the identification of [p] and [~ ~ p], and might perhaps generalize to further cases. Drawing on a famous passage from Ramsey, we are asked to imagine a language that does not have a symbol for negation, but instead turns the sentence ‘p’ (or symbols composing it) upside down for expressing that ~ p. Dorr writes:

If we spoke Ramsey’s imagined language, we would simply have no pairs of distinct formulae in our language that relate to one another in the same way that ‘p’ relates to ‘~~p’ in our actual language. If there are truths of the form ‘~(p≈ ~~p)’, they are inexpressible in such a language. But it is hard to believe that the use of such a language would be any sort of a handicap from a metaphysical point of view. (2016: 63 [Symbolism adapted])

If we take this kind of consideration where it leads, it seems many disputes between worldly ground-theorists can be settled. For instead of the Ramsey language, consider a sentential language with only the Sheffer stroke ‘↑’, where ‘p↑q’ is true if and only if ‘p’ and ‘q’ are not both true. Suppose that, for whatever reason, this language turns out to be blind to any truth expressed otherwise by ‘ ~ (~ (p∧q) ≈ ~ p∨ ~ q))’. (If e.g. we identify contents with truth-functions expressed, both sentences flanking the non-equivalence are equivalent in content to ‘p↑q’.) The claim that such a truth is *inexpressible* in this language is much too strong in this case, as the need for the supposition attests.^27^ Nonetheless—one might continue–, it is hard to believe that the use of such a language, paired with the supposition, would be any sort of a handicap from a metaphysical point of view. Now cases of this sort *are* disputed—indeed the logics of worldly ground proposed by Correia deny the *De Morgan* equivalences. The kind of consideration put forward by Dorr is only helpful for solving such cases if paired with some sort of criterion for what would constitute ‘a handicap from a metaphysical point of view’ that would drive a wedge between the Ramsey and these Sheffer languages. Once again, the dispute is only pushed back a step.^28^

If these considerations are on the right track, it seems advisable to keep both aspects of the distinction between so-called *worldly* and *representational* conceptions of ground apart. The more manageable dispute concerns the *individuation* of grounds and groundees. Whether they should be conceived as entities ‘out there’ or as representational entities in some sense is orthogonal to the former question. I do not deny that the second aspect of this distinction is substantial, nor do I suggest that groundhogs should avoid taking a stance on it. In fact, allowing for fine-grained individuation of worldly entities makes the debate between different conceptions of ground more engaging. For example, it would be strange to claim that conceptions of ground with distinct individuation criteria for relata are equally valid while assuming that ground relates to the same worldly entities, such as facts, in both conceptions.

Since the present proposal only involves the combination of states, it offers the advantage of neutrality regarding the question that began this discussion. Whether states and their combinations reflect how the world is or how we represent it, the semantics performs well on the two assignments: it gives an informative answer to how finely grounds and groundees are individuated; and a relatively simple answer, close to the one provided in the so-called ‘worldly’ case, to the question of what ground-theoretical claims are sensitive to, that is to verifiers and how they are combined into further states.

## Footnotes

1 I use ‘relata’ and ‘entities’ in a broader sense than usual, since they should also apply to grounds and groundees under an approach that takes grounding to be expressed by an operator rather than a relational predicate.

2 I hasten to add that the latter does *not* correspond to Fine’s own notion of conjunctive part (see Fine 2017a: 649). Perhaps the best label for the notion defined in the main text would be ‘*proper* conjunctive part’. I stick to the simpler label for the sake of exposition.

3 ‘Takes part in’ expresses being amongst members of a plurality.

4 Improper parthood is defined in terms of fusion thus: ∀s∀t (s⊑t ↔ _df_ s⊔t = t). Using this, we define proper parthood thus: ∀s∀t (s⊏t ↔ _df_ s⊑ t ∧ s ≠ t).

5 See footnote 2.

6 This indeed holds in general for representational accounts, while only restrictively in worldly accounts for reasons already discussed.

7 In effect, this boils down to the fact that conjunctive parthood entails weak partial grounding, but the converse does not hold (with respect to a suitable space of propositions).

8 Notice that this kind of example holds irrespectively of the inclusive treatment of verification of disjunctions.

9 I thank an anonymous referee for pressing me to be explicit about this.

10 The label ‘Idempotency’ is usually used for a less general principle, namely that s⊔s = s. I borrow the label of the more general principle from Cotnoir (2015).

11 That it operates on sequences is required for combination not to obey *Absorption*, that is, that + (s, s, …) =  + (s,…). Another option would be to take it to operate on multi-sets. I choose the former for simplicity. The restriction to finite sequences is due to the following observation: let (s, s,…) be the sequence in which ‘s’ occurs countably many times. Then + (s, + (s, s,…)) ≠  + (s, s,…) according to *Alterpotency*; but + (s, + (s, s,…)) =  + (s, s,…) per *Leveling* (and standard set-theory). Presumably infinitary resources, or frameworks alternative to standard set theory might allow us to drop this restriction. However, since the semantics is mainly used for finitary languages, the restriction is harmless. See Vogt & Werner (2024) for independent motivation of this restriction. I thank an anonymous referee for helpful discussion on these points.

12 Of course, sequence formation does not deliver a single result applied to a plurality; given the convention stated, what holds for the sequence explicitly given should also hold for every permutation of ist members, as per *Permutation*.

13 Reflexivity and transitivity are ensured by the definition. The condition laid down by Fine (2010: 568) for anti-symmetry, namely: *Anti-cyclicity*: If x =  + (…, + (…,x,…),…) then x =  + (…,x,…); is satisfied since the antecedent never holds due to *Alterpotency*.

14 Consideration of combinations of no states is required for some applications having to do with zero-ground (Fine 2012a).

15 DeRosset & Fine (2023) also allow for combination to apply to a single state; as they notice, the role the unary case plays in their account is similar to that of the operation of ‘raising’ in Krämer’s (2018) account. More on these accounts below.

16 + *Commutativity*: s + t = t + s; + *Associativity*: (s + t) + u = s + (t + u). (Where ‘s + t’ is a notational variant for ‘ + (s,t)’.).

17 This is stronger than the denial that there are propositions closed under combination, where a proposition is thus closed whenever for every sequence x of states in the set of verifiers or falsifiers, there is a state s in that same set such that + (x) = s.

18 I illustrate with the proof of (1). Suppose s ∣⊢ P. Then, since s⊏_+_  + (s) and + (s) ∣⊢ P∨P, P ⊂ _c_ P∨P. Given that P is not unbounded, there is a verifier s of P and a verifier + (s) of P∨P which is not a verifier of P, such that for every verifier t of P: ~ (+ (s)⊏_+_ t). Hence it is not the case that P∨P ⊂ _c_ P. Hence P ≺ P∨P. By the clause for verification of disjunctions, + (s) ∣⊢ P∨P, for every s such that s ∣⊢P. Thus P < P∨P. The proofs of (2) and (3) are analogous, but use the fact that + (s,s) ∣⊢ P∨P, for s ∣⊢P, by the clause for disjunction verification; and the fact that + (s,s) ∣⊢ P∧P, for s ∣⊢P, by the clause for conjunction verification, respectively.

19 Proof: (4) Suppose s∣⊢P. A verifier of ~  ~ P is either a falsifier of ~ P, i.e. a verifier of P; or a combination of one or two of such verifiers. Given that s⊏_+_  + (s), every verifier of P is a proper part of a verifier of ~  ~ P, i.e. P ⊂ _c_ ~  ~ P. Given that P is not unbounded, there is a verifier s of P and a verifier + (s) of ~  ~ P which is not a verifier of P, such that for every verifier t of P: ~ (+ (s)⊏_+_ t). Hence it is not the case that ~  ~ P ⊂ _c_ P. Hence P ≺ ~  ~ P. Since by the clauses + (s) ∣⊢ ~  ~ P whenever s∣⊢P, we have it that P <  ~  ~ P. The proof of (5) is analogous.

20 Why binary, that is, why stop at (sequences of) 2 states in the clause for negation? In principle, one could adopt the more general clause: s ∣⊢ ~ P iff s ⊣∣ P; or s =  + (t_1_, …, t_n_) such that t_1_ ⊣∣ P and t_2_ ⊣∣ P and…and t_n_ ⊣∣ P. This would correspond to a generalized Peirce arrow, applying to any finite number of sentences. While this would not make any difference for the language with binary conjunction, disjunction and unary negation, it might make for a difference in languages equipped with, for instance, negations of diverse arities. For simplicity and in view of the more usual binary case, I stick to the clause of the main text. I thank an anonymous referee for helpful discussion.

21 While of course required for a more thorough appreciation of the present approach, soundness and (ideally) completeness proofs with respect to logics of representational ground must also be left for another occasion.

22 Similar considerations would presumably apply to slots conceived as positions (not necessarily space-like), or to a related conception of fusion as applying to parts *in a certain order*.

23 An alternative view would have it that there is *one* role that is occupied by *a* twice: once *with respect to* b; and once *with respect to* c. This notion of relative occupation would call for a different individuation of wholes, but the same points in the main text would apply with modifications in the details.

24 The present treatment of combination generates a junky mereology, i.e. one in which every set of states might be combined into a larger whole. Whether junky mereologies enjoy independent motivation is a matter of dispute. See Bohn (2009) and Mormann (2014) for defenses; Vogt & Werner (2024) for recent discussion.

25 Correia defines a measure of logical complexity for which it holds that every ground has strictly less complexity than its consequences. (Correia 2017: section 3.1.) The strict increase of complexity from grounds to consequences might be maintained in our account if the measure takes into consideration the scope to which negation applies: that is, it it assigns a greater complexity to ~ (P∧Q) than for instance to ~ P∧ ~ Q.

26 Notice that non-inclusive disjunction here at stake should not be equated with exclusive disjunction in the usual sense. For truth of the non-inclusive disjunction is compatible with both disjuncts obtaining: the crucial point is that it is not verified by both of them together.

27 In the effort of translation from a language with ‘∧’, ‘∨’ and ‘ ~ ’, one might for instance define independently ‘ ~ p’ as ‘p↑p’; ‘p∧q’ as ‘(p↑q)↑(p↑q)’; and ‘p∨q’ as ‘(p↑p)↑(q↑q)’. Then, if one starts off from the ultimate subformulas, ‘ ~ (p∧q)’ and ‘ ~ (~ p∨~ q)’ are *not* translated into the same formula of the Sheffer-language. I thank an anonymous referee for pertinent discussion.

28 One might also consider variants of the Ramsey language in which, instead of turning a sentential symbol upside down, one rotates it by some constant angle, e.g. 90 degrees clockwise. ‘ ~ (p≈ ~  ~  ~  ~ p)’ is then not expressible in this language, though ‘ ~ (p≈ ~  ~ p)’ is. Surely, these languages are weird, but it is far from clear that their weirdness touches on metaphysical matters. I hasten to add that this application might not have been Dorr’s original intent with the argument.

## References

[CR1] Bennett, K. (2013). Having a part twice over. *Australasian Journal of Philosophy,**91*(1), 83–103.

[CR2] Bohn, E. S. (2009). An argument against the necessity of unrestricted composition. *Analysis,**69*(1), 27–31.

[CR3] Correia, F. (2010). Grounding and Truth-Functions. *Logique Et Analyse,**53*(211), 251–279.

[CR4] Correia, F. (2016). On the logic of factual equivalence. *Review of Symbolic Logic,**9*(1), 103–122.

[CR5] Correia, F. (2017). An impure logic of representational grounding. *Journal of Philosophical Logic,**46*(5), 507–538.

[CR6] Correia, F. (2020). Granularity. In M. J. Raven (Ed.), *The Routledge handbook of metaphysical grounding* (pp. 228–243). Routledge.

[CR7] Cotnoir, A. J. (2015). Abelian mereology. *Logic and Logical Philosophy,**24*(4), 429–447.

[CR8] DeRosset, L., & Fine, K. (2023). A Semantics for the Impure Logic of Ground. *Journal of Philosophical Logic,**52*(2), 415–493.

[CR9] Dorr, C. (2016). To Be F Is To Be G. *Philosophical Perspectives,**30*(1), 39–134.

[CR10] Fine, K. (2000). Neutral relations. *Philosophical Review,**109*(1), 1–33.

[CR11] Fine, K. (2010). Towards a theory of part. *Journal of Philosophy,**107*(11), 559–589.

[CR12] Fine, K. (2012a). Guide to ground. In F. Correia & B. Schnieder (Eds.), *Metaphysical grounding* (pp. 37–80). Cambridge University Press.

[CR13] Fine, K. (2012b). The pure logic of ground. *Review of Symbolic Logic,**5*(1), 1–25.

[CR14] Fine, K. (2017a). A theory of truthmaker content I: Conjunction, disjunction and negation. *Journal of Philosophical Logic,**46*(6), 625–674.

[CR15] Fine, K. (2017b). A theory of truthmaker content II: Subject-matter, common content, remainder and ground. *Journal of Philosophical Logic,**46*(6), 675–702.

[CR17] Krämer, S. (2018). Towards a theory of ground-theoretic content. *Synthese,**195*(2), 785–814.

[CR18] Krämer, S. (2019). Ground-theoretic equivalence. *Synthese,**198*(2), 1643–1683.

[CR19] Krämer, S., & Roski, S. (2015). A note on the logic of worldly ground. *Thought: A Journal of Philosophy,**4*(1), 59–68.

[CR20] Mormann, T. (2014). Set theory, topology, and the possibility of junky worlds. *Notre Dame Journal of Formal Logic,**55*(1), 79–90.

[CR21] Raven, M. J. (Ed.). (2020). *The Routledge handbook of metaphysical grounding*. Routledge.

[CR22] Schnieder, B. (2011). A logic for ‘because.’ *Review of Symbolic Logic,**4*(3), 445–465.

[CR23] Vogt, L., & Werner, J. (2024). Cardinal composition. *Erkenntnis,**89*(4), 1457–1479. 38616867 10.1007/s10670-022-00591-1 PMC11014879

